# Exploring Emotion Regulation Tactics in Younger and Older Adults Using Virtual Reality

**DOI:** 10.1093/geroni/igaf122.3275

**Published:** 2025-12-31

**Authors:** Jasmine Moon, Blake Ebright-Jones, Samantha Furey, Derek Isaacowitz

**Affiliations:** Washington University in St. Louis, St. Louis, Missouri, United States; Washington University in St. Louis, St. Louis, Missouri, United States; Washington University in St. Louis, St. Louis, Missouri, United States; Washington University in St. Louis, St. Louis, Missouri, United States

## Abstract

Older adults typically report higher levels of emotional well-being compared to younger adults, and emotion regulation may play a key role. While past research investigating age differences in emotion regulation strategy selection has been inconsistent, recent everyday life studies have identified age differences in emotion regulation tactics. Tactics refer to the ways in which individuals implement strategies and can be operationalized in terms of valence and direction: positive-approaching, negative-approaching, and negative-receding. Most tactic studies have used experience sampling, but Virtual Reality (VR) can be used to investigate age differences in an immersive setting that mimics real-life environments, allowing for emotional responses to more controlled contexts. As the first study to examine age differences in tactics within VR, the current study had participants (N = 70; ages 18-79) first interact with a negatively-valenced virtual environment for three minutes. Then, participants reported whether they had regulated their emotions while in the simulation, and if so, which regulation strategy and tactic they had used to regulate their emotions. Participants were also asked whether they thought they were successful in their regulation attempts. Younger adults were more likely to report regulation success compared to older adults, but no other group differences were significant. This finding contrasts with results from traditional lab tasks and everyday life assessments, suggesting VR offers a promising avenue to further investigate individual differences and environmental influences on real-world emotion regulation across the lifespan.

